# An immunological mechanism of resistance to CDK4/6 inhibitors in HR^+^ breast cancer

**DOI:** 10.1080/2162402X.2025.2520269

**Published:** 2025-07-15

**Authors:** Claudia Galassi, Giulia Petroni, Simon R.V. Knott, Lorenzo Galluzzi

**Affiliations:** Department of Pharmacology, Weill Cornell Medical College, New York, NY, USA; Department of Experimental and Clinical Medicine, University of Florence, Florence, Italy; Department of Biomedical Sciences, Cedars-Sinai Medical Center, Los Angeles, CA, USA; Cancer Signaling and Microenvironment Program, Fox Chase Cancer Center, Philadelphia, PA, USA

**Keywords:** Circulating biomarker, endocrine therapy, hypoxia, palbociclib, single-cell RNA sequencing, TAMs

## Abstract

CDK4/6 inhibitors are central to the clinical management of HR^+^HER2^−^ breast cancer. We have recently demonstrated that immunosuppressive, IL17-secreting γδ T cells recruited to the tumor microenvironment by a CCL2-dependent mechanism upon CDK4/6 inhibition can repolarize tumor-associated macrophages toward a CX3CR1^+^ phenotype associated with resistance to therapy.

Cyclin-dependent kinase 4/6 (CDK4/6) inhibitors are currently employed together with endocrine therapy (ET) as a first-line intervention in women with advanced/metastatic HR^+^HER2^−^ breast cancer.^1^ CDK4/6 inhibitors have indeed demonstrated consistent progression-free survival (PFS) benefits across multiple clinical trials enrolling this patient population, although less consistent results with respect to overall survival (OS), at least in some cases owing to the emergence of adaptive resistance to treatment.^1^ Mechanistically, CDK4/6 inhibitors have been conceived to operate by enforcing a stable proliferative arrest via retinoblastoma 1 (RB1) and the so-called DREAM complex, overall preventing the activation of E2F-dependent transcriptional programs that promote the G_1_-S cell cycle transition.^2^ In line with this notion, common mechanisms of resistance to CDK4/6 inhibitors include genetic *RB1* losses as well as defects in the tumor protein 53 (TP53, best known as p53) system, which is important to suppress compensatory CDK2 activation in the context of efficient CDK4/6 blockage.^1^

That said, CDK4/6 inhibitors have also been shown to mediate immunostimulatory effects that may (at least partially) contribute to their clinical efficacy.^3,4^ Specifically, CDK4/6 inhibitors appear to: (2) stimulate the secretion of immunostimulatory cytokines such as type III interferon (IFN) and chemokines such as C-C motif chemokine ligand 2 (CCL2) by malignant cells, resulting in the recruitment of immune effector cells to the tumor microenvironment (TME) and their activation; (2) promote MHC Class I exposure on the surface of cancer cells, hence rendering them increasingly visible to the host immune system; and (3) mediate a direct antiproliferative effect on immunosuppressive CD4^+^CD25^+^FOXP3^+^ regulatory T (T_REG_) cells.^3,5^ In this context, we set to investigate whether (at least in some circumstances) resistance to CDK4/6 inhibitors would also involve local or systemic immunosuppression. Harnessing an immunocompetent model of HR^+^ mammary carcinogenesis that recapitulates key immunobiological features of its human counterpart,^6^ as well as clinical samples from no less than 6 distinct cohorts of patients with HR^+^HER2^−^ breast cancer, we have recently identified a novel immunological mechanism of adaptive resistance to CDK4/6 inhibitors that involves interleukin 17 (IL17)-secreting γδ T cells and C-X3-C motif chemokine receptor 1 (CX3CR1)-expressing tumor-associated macrophages (TAMs).^7^

We set to investigate immunological changes in the tumor microenvironment (TME) of female C57BL/6 mice bearing mammary carcinomas as driven by slow-release medroxyprogesterone acetate (MPA, M) pellets and oral dimethylbenz[α]anthracene (DMBA, D)^6^ receiving the prototypic CDK4/6 inhibitor palbociclib (P) plus tamoxifen (T)-based ET, optionally preceded by focal hypofractionated radiation therapy (RT) in 3 fractions of 10 Gy each. These tumors are poorly sensitive to immune checkpoint inhibitors,^6^ but respond to palbociclib, a therapeutic activity that can be increased when RT is delivered before P (RT→P).^8^ Single-cell RNA sequencing (scRNAseq) revealed that P+T elicited the accumulation of γδ T cells in the TME of M/D-driven tumors, an effect that could not be documented in lesions subjected to RT→P+T. These γδ T cells exhibited a transcriptional profile that had previously been associated with immunosuppressive properties,^9^ notably *Il17a* expression coupled with reduced interferon gamma (*Ifng*) and *Cd27* levels. Similar immunosuppressive features could be documented by scRNAseq in γδ T cells from human HR^+^HER2^−^ breast cancer samples. In line with the ability of γδ T cells to promote resistance to CDK4/6 inhibitors, the therapeutic activity of P+T against M/D-driven mammary tumors could be improved by: (1) a γδ TCR-antagonistic antibody, (2) an IL17A-neutralizing antibody, and (3) the deletion of *Tcrd* (which encodes one of the γδ TCR chains)^10^ from the host.^7^

Our mouse scRNAseq dataset as well as bioinformatic analyses comparing patients with ER^+^HER2^−^ breast cancer from the public METABRIC dataset^11^ based on transcriptional signatures of IL17 signaling pointed to CCL2 and its cognate receptor C-C motif chemokine receptor 2 (CCR2) as to potential drivers of γδ T cell infiltration in M/D-driven tumors responding to P+T. Indeed, both mouse and human HR^+^ breast cancer cells secreted CCL2 in response to P. Moreover, CCL2 neutralization with a specific monoclonal antibody not only improved the therapeutic effects of P+T against M/D-driven mammary carcinomas, but also limited their infiltration by IL17-producing γδ T cells. Interestingly, it turned out that the ability of RT to prevent the recruitment of γδ T cells by P+T reflects its capacity to select (at least initially) for hypoxic tumor regions that (1) are inhospitable for γδ T cells,^12^ and (2) interfere with P+T-driven CCL2 secretion by cancer cells.^7^

Mouse scRNAseq data linked γδ T cell recruitment as elicited by P+T to an enrichment in CX3CR1^+^ TAMs, which (compared to their CX3CR1^−^ counterparts) exhibited a transcriptional profile associated with immunosuppression, notably the downregulation of genetic signatures of type I IFN and IFNG signaling, as well as (1) upregulation of the interleukin 17 receptor A (*Il17ra*), and (2) an increased abundance of transcripts involved in IL17 signaling. Similar transcriptional features could be documented in CX3CR1^+^ TAMs from human HR^+^HER2^−^ breast cancer samples. Of note, repolarizing CX3CR1^+^ TAMs toward an immunostimulatory configuration with a monoclonal antibody targeting colony stimulating factor 1 receptor (CSF1R) ameliorated the activity of P+T against M/D-driven mammary carcinoma, to a magnitude similar to IL17A neutralization. Blocking CSF1R and neutralizing IL17A exhibited no epistatic interactions with respect to the therapeutic efficacy of P+T, suggesting that these two processes operate within the same resistance mechanism.^7^

Importantly, genetic signatures of γδ T cell infiltration or IL17 signaling in diagnostic biopsies from patients with 1,042 HR^+^HER2^−^ breast cancer from the METABRIC dataset^11^ were associated with decreased disease-specific survival (DSS). Moreover, γδ T cells were enriched in the microenvironment of human grade III *vs* I or II HR^+^HER2^−^ breast cancer, as documented in diagnostic biopsies from 86 patients. Along similar lines, an increased abundance of CX3CR1^+^ TAMs at baseline was associated with a lack of pathological complete response (pCR) in 12 patients enrolled in a clinical trial testing neoadjuvant pembrolizumab followed by pembrolizumab plus stereotactic body RT (SBRT) in patients with high-risk HR^+^HER2^−^ breast cancer.^13^ Finally, (1) an increased amount of activated γδ T cells in the circulation of 23 patients with HR^+^HER2^−^ breast cancer prospectively allocated to CDK4/6 inhibitors was associated with decreased PFS; (2) in the same cohort, responders (but not non-responders) exhibited an early increase in circulating CCL2; (3) in 8 patients with HR^+^HER2^−^ breast cancer, relapse after CDK4/6 inhibition was linked with an increase in intratumoral γδ T cells.^7^

In summary, our data delineate a novel immunological mechanism through which γδ T cells recruited to the TME of HR^+^HER2^−^ mammary tumors upon CCL2 secretion as driven by CDK4/6 inhibitors promote the IL17-dependent repolarization of TAMs toward a CX3CR1^+^ profile associated with resistance to therapy (Figure 1). As this mechanism can be averted by focal hypofractionated RT delivered prior to P+T, a randomized, Phase II clinical trial (CIMER, NCT04563507) is currently recruiting subjects to investigate standard-of-care CDK4/6 inhibition plus ET *vs* SBRT followed by CDK4/6 inhibition plus ET in patients with oligometastatic (≤5 metastatic sites, no brain involvement) HR^+^HER2^−^ breast cancer. As a future development, it will be interesting to understand whether IL17 inhibitors (no less than 3 of which are currently approved for use in patients with psoriasis and other inflammatory conditions)^14^ or CSF1R inhibitors such as pexidartinib and vimseltinib (which are currently approved for use in patients with tenosynovial giant cell tumors)^15,16^ can be safely and effectively combined with CDK4/6 inhibitors in patients with HR^+^HER2^−^ breast cancer that are not eligible to SBRT.

**Figure 1.**
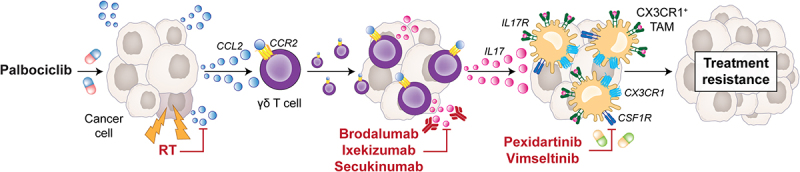
A new immunological mechanism of resistance to CDK4/6 inhibitors in HR^+^HER2^−^ breast cancer. At least in an immunocompetent mouse model of HR^+^HER2^−^ breast cancer, the therapeutic effects of the CDK4/6 inhibitor palbociclib appear to be limited by a mechanism involving: (1) CCL2 secretion by malignant cells; (2) CCL2-dependent recruitment of γδ T cells to the tumor microenvironment; (3) γδ T cell secretion of IL17; and (4) IL17-dependent enrichment of tumor-associated macrophages (TAMs) toward an immunosuppressive CX3CR1^+^ profile. By virtue of its ability to (at least initially) select for hypoxic, CCL2-incompetent tumor regions, hypofractionated radiation therapy (RT) may be effectively used to avert this resistance mechanism, hence representing a promising therapeutic partner for CDK4/6 inhibitors in the clinic. Whether blocking IL17 or repolarizing CX3CR1^+^ TAMs toward an immunostimulatory state with CSF1R inhibitors may also constitute clinically viable approaches to limit resistance to CDK4/6 blockers in patients with advanced/metastatic HR^+^HER2^−^ breast cancer remains to be formally elucidated.
